# Exploring the Relationship Between Blood Transfusions and Development of Bronchopulmonary Dysplasia in Neonates

**DOI:** 10.7759/cureus.80706

**Published:** 2025-03-17

**Authors:** Abdulrahman Al-Matary, Ibrahim AlShalan, Fawaz M AlDhafiri, Munthir Almujahid, Abdulrahman Almazyad

**Affiliations:** 1 Department of Neonatology, Health Science Centre, Winnipeg, CAN; 2 Neonatology, King Fahad Medical City, Riyadh, SAU; 3 Pedatrics, King Fahad Medical City, Riyadh, SAU; 4 Pediatrics, King Fahad Medical City, Riyadh, SAU

**Keywords:** bronchopulmonary dysplasia (bpd), : neonates, newborn blood transfusion, packed red blood cell transfusion, preterm infants

## Abstract

Background: Transfusions of red blood cells and platelets may worsen pulmonary inflammation and contribute to the development of bronchopulmonary dysplasia (BPD), a common lung condition in preterm infants. Although nearly all infants with severe BPD have received transfusions, their role as a potential cause of BPD has not been thoroughly studied.

Objectives: This study aimed to explore the relationship between blood product transfusions and the development of BPD among preterm neonates in the Department of Neonatology at King Fahad Medical City, Riyadh, Saudi Arabia.

Methods: A retrospective study was conducted from 2011 to 2020 on neonates with a gestational age of less than 32 weeks who were admitted to the hospital within 48 hours of birth. Data were extracted from the department's medical records on patient demographics, clinical factors, and blood transfusions. Logistic regression analysis was performed to assess the relationship between blood transfusion and the development of BPD in the study cohort.

Results: A total of 1,553 neonates were included in the study. The mean gestational age was 28.8 ± 2.7 weeks, and the mean birth weight was 1264.2 ± 515.1 grams. Among the neonates, 183 (11.8%) were diagnosed with BPD. Neonates who received blood transfusions had a significantly higher likelihood of developing BPD compared to those who did not (OR = 9.1, 95% CI = 6.3-13.1), with the risk being even higher among those who received fresh frozen plasma (OR = 9.9). After adjusting for potential confounders, multivariate logistic regression analysis confirmed that blood transfusion remained a significant factor in the development of BPD (OR = 3.8, 95% CI = 2.5-5.8). Stepwise regression analysis further identified blood transfusion as the strongest predictor of BPD (OR = 4.5, 95% CI = 2.91-6.70). Additional significant predictors included retinopathy of prematurity (ROP), patent ductus arteriosus (PDA), sepsis, and non-invasive ventilation (NIV).

Conclusion: This study found a significant association between blood transfusions and the development of BPD in preterm neonates, and it was found as a strong predictor. Other factors such as ROP, PDA, sepsis, and NIV use were also associated with BPD. The findings suggest that blood transfusion may play a critical role in the development of BPD.

## Introduction

Bronchopulmonary dysplasia (BPD) is a frequent and significant complication in premature infants, particularly those with low birth weights (BW) and gestational ages (GA) [1]. BPD develops as a consequence of inflammation, oxygen exposure, and mechanical ventilation on the immature lungs, although its pathophysiology is not fully understood [2]. This condition is linked to long-term respiratory issues such as asthma, wheezing, reduced exercise tolerance, and recurrent lower respiratory tract infections [3]. In addition, comorbidities like sepsis, necrotizing enterocolitis, pulmonary hemorrhage, and maternal factors such as smoking, chorioamnionitis, male sex, oxygen therapy, and prolonged mechanical ventilation have all been shown to contribute to the development of BPD [4].

Due to the lack of a universally accepted classification for BPD, there is considerable variability in prenatal procedures, care approaches, and prevalence rates of BPD across different centers. The highest documented rates of BPD are found in infants with very low BW and early GA [5]. According to data from the Neonatal Research Network, the incidence of BPD in extremely low birth weight infants (birth weight less than 1500 grams) ranges from 40% to 68%, depending on the diagnostic criteria used. This occurrence is inversely related to the infant’s GA, with a higher risk in those born at earlier gestational ages [6].

Anemia remains a common concern in neonatal intensive care units, particularly among preterm infants, who are more susceptible to it than full-term babies [7]. The pathophysiology of neonatal anemia is complex, involving low iron stores, reduced red blood cell (RBC) production, increased RBC destruction, shortened RBC lifespan, and blood loss due to clinical procedures [8]. Neonatologists often decide to administer blood transfusions when they believe the benefits outweigh the risks, though these decisions are often based more on clinical experience and local guidelines than on evidence-based data [9].

Blood transfusions, particularly of RBCs and platelets, have been shown to potentially induce or worsen pulmonary inflammation [10-11]. Developmental differences between neonatal and adult blood components suggest that transfused adult platelets and RBCs may be more pro-inflammatory in neonates than in adults, potentially contributing to the inflammatory processes associated with BPD [12-14]. Most neonates with severe BPD have had multiple transfusions, but the role of blood transfusions in the development of BPD remains under-researched [15]. Given that transfusions of RBCs and platelets can exacerbate pulmonary inflammation, and considering that many infants with severe BPD have received multiple transfusions, it is essential to explore whether blood transfusions contribute to the onset or worsening of BPD.

Thus, the main objective of this study is to investigate the relationship between blood product transfusions, specifically red blood cells, platelets and fresh frozen plasma, and the development of BPD in neonates delivered at King Fahad Medical City in Riyadh, Saudi Arabia. Moreover, the study aims to determine whether blood transfusions contribute to the increased risk of BPD, accounting for potential confounding factors such as gestational age, birth weight, and medical conditions (e.g., sepsis, patent ductus arteriosus, necrotizing enterocolitis, periventricular leukomalacia, intraventricular hemorrhage, retinopathy of prematurity, BPD). These findings could provide new insights into the potential risks associated with blood transfusions in neonates, addressing a gap in the current literature where the relationship between transfusions and BPD is understudied.

## Materials and methods

This retrospective study was conducted in the Department of Neonatology at King Fahad Medical City, Riyadh, Saudi Arabia, using data collected from January 1, 2011, to December 31, 2020, from the department's medical records.

The study included neonates who met specific criteria: (1) newborns with a gestational age of less than 32 weeks and (2) those admitted to the hospital within 48 hours of birth. Infants were excluded if they met any of the following conditions: (1) severe ischemic hypoxic encephalopathy and chromosomal abnormalities; (2) upper respiratory tract abnormalities, lung malformations, or pulmonary hypoplasia; (3) genetic mutations related to the respiratory system's panel gene; (4) death within 14 days or discontinuation of treatment before reaching 36 weeks corrected gestational age; or (5) failure to receive critical clinical information.

The study subjects were divided into two groups according to the main independent variable: Group 1, which included patients who did not receive transfusions, and Group 2, which included those who received transfusions before reaching 36 weeks corrected gestational age.

For this study, several medical conditions were defined: the outcome variable; BPD was identified by the requirement for oxygen at 36 weeks of gestational age [16]. Sepsis was diagnosed when systemic symptoms were present along with a positive blood culture, with early-onset sepsis occurring within the first seven days and late-onset sepsis occurring later [17]. Necrotizing enterocolitis (NEC) was diagnosed based on systemic symptoms and radiographic findings, with its severity assessed using the modified staging criteria of Bell et al. [18]. Patent ductus arteriosus (PDA) was diagnosed through echocardiography and clinical symptoms [19], while retinopathy of prematurity (ROP) was diagnosed by a neonatologist and confirmed by an ophthalmologist following the American Academy of Ophthalmology's schedule [20]. Periventricular leukomalacia (PVL) was characterized by white matter loss in the brain due to insufficient blood or oxygen supply to the periventricular region [21], and intraventricular hemorrhage (IVH) was diagnosed based on bleeding in the brain's ventricular system, potentially caused by trauma or hemorrhagic stroke [22].

Data were collected by trained study workers from the patients' medical records, including gestational age, birth weight, gender, Apgar scores at one and five minutes, length of stay (LOS), mode of ventilation, and any neonatal anomalies.

Statistical analyses were conducted using IBM SPSS Statistics for Windows, Version 22.0 (released 2013, IBM Corp., Armonk, NY). Descriptive statistics were used, with categorical data presented as frequencies and percentages, and continuous data presented as means and standard deviations (SD). To compare the neonates based on their BPD status and characteristics, including whether they received blood transfusions, a chi-square test was used for categorical variables, and a Student's t-test was applied for continuous variables. Univariate and multivariate logistic regression models were employed to assess the association between blood transfusion and BPD, controlling for potential covariates. The results were expressed as odds ratios (OR) with 95% confidence intervals. Additionally, a stepwise logistic regression was performed to identify the most significant factors associated with BPD, using a p-value of 0.10 as the inclusion criterion and 0.20 as the exclusion criterion. 

Since the study was retrospective and the data had already been collected as part of routine clinical practice and de-identified, no ethical review was required. However, the study protocol was approved by the Ethical Committee of King Fahad Medical City in Riyadh, Saudi Arabia on May 28, 2024 (IRB log number: 24-275). Data were obtained from our neonatal database, and no human subjects were included in the study.

## Results

The study included a total of 1,553 neonates: 734 females (47.3%) and 819 males (52.7%) (Table 1). The mean gestational age was 28.8 ± 2.7 weeks, with a majority (59.8%) born at >28 weeks gestation. The mean birth weight was 1264.2 ± 515.1 grams and majority of neonates had birth weights >1500 g (30.0%). Apgar scores at one minute and five minutes were 5.6 ± 2.1 and 7.6 ± 1.6, respectively. In terms of ventilation modes, most neonates were treated with synchronized intermittent mandatory ventilation (SIMV) (45.9%) and non-invasive ventilation (NIV) (43.9%). Regarding medical conditions, sepsis was observed in 20.2% of neonates, while other conditions included PDA (14.8%), NEC (3.7%), PVL (2.6%), IVH (5.7%), ROP (5.6%), and BPD (11.8%). Blood transfusions were administered to 31.6% of the neonates, with 18.1% receiving complete blood, 8.3% receiving platelets, and 5.2% receiving fresh frozen plasma. The average length of hospital stay was 41.9 ± 41.3 days, and 20.4% of the neonates in the study sample died. However, the overall death rate at the studied center during the study period was 0.7% (317 out of 44,566 neonates).

**Table 1:** Characteristics of the studied neonates

Characteristics	Description	N (%)
Sex	Male	819 (52.7)
	Female	734 (47.3)
Gestational age (weeks)	mean ± SD	28.8 ± 2.7
Gestational age	≤ 28 weeks	624 (40.2)
	> 28 weeks	929 (59.8)
Birth weight (gm)	mean ± SD	1264.2 ± 515.1
	< 750	237 (15.3)
Birth weight (gm)	750-<1000	272 (17.5)
	1000- <1250	288 (18.5)
	1250-1500	290 (18.7)
	>1500	466 (30.0)
Apgar Score	Apgar 1 (mean ± SD)	5.6 ± 2.1
	Apgar 5 (mean ± SD)	7.6 ± 1.6
	Synchronized intermittent mandatory ventilation (SIMV)	713 (45.9)
Mode of ventilation	Non-invasive ventilation (NIV)	681 (43.9)
	High frequency oscillatory ventilation (HFOV)	159 (10.2)
	Sepsis	313 (20.2)
	Patent Ductus arteriosus (PDA)	230 (14.8)
Medical conditions	Necrotizing enterocolitis	57 (3.7)
	Periventricular leukomalacia (PVL)	41 (2.6)
	Intraventricular hemorrhage (IVH)	89 (5.7)
	Retinopathy of prematurity (ROP)	87 (5.6)
	Bronchopulmonary dysplasia (BPD)	183 (11.8)
	No	1063 (68.4)
Blood transfusion	Complete blood	281 (18.1)
	Platelets	129 (8.3)
	Fresh frozen plasma (FFP)	80 (5.2)
Hospitalization and patient outcomes	Length of hospital stay (days) mean ± SD	41.9 ± 41.3
	Death	317 (20.4)

Table 2 compares the neonates with and without BPD. Among the 183 neonates with BPD and the 1,370 without, the male-to-female ratio was slightly higher in the BPD group (57.9% males) compared to the non-BPD group (52.0% males), although not statistically significant (p = 0.14). The BPD group had a significantly higher proportion of neonates born at or before 28 weeks of gestation (70.5%) compared to the non-BPD group (36.1%) (p < 0.0001). Similarly, a higher percentage of neonates with BPD had lower birth weights, with 32.2% weighing less than 750 grams, compared to 13.0% in the non-BPD group, and 34.4% of BPD neonates weighed between 750 and 999 grams, compared to 15.3% in the non-BPD group (p < 0.0001). Apgar scores at one minute (4.9 ± 2.0 vs. 5.7 ± 2.1) and five minutes (7.2 ± 1.5 vs. 7.7 ± 1.6) were significantly lower in the BPD group (p < 0.0001 and p = 0.002, respectively). The mode of ventilation also differed significantly, with a higher percentage of BPD neonates receiving SIMV (71.6%) compared to the non-BPD group (42.5%) and fewer BPD neonates receiving NIV (12.0%) (p < 0.0001). Sepsis, PDA, NEC, and ROP was significantly higher in the BPD group (p < 0.0001 for all). However, PVL and IVH were more prevalent in the BPD group, although these differences were not statistically significant (p = 0.12 and p = 0.13, respectively). Blood transfusions were significantly more common in the BPD group, with 44.3% receiving complete blood, compared to just 14.6% in the non-BPD group (p < 0.0001). The length of hospital stay was significantly longer for neonates with BPD (107 ± 48.0 days) compared to those without (33.2 ± 31.4 days) (p < 0.0001). Finally, the death rate was significantly lower in BPD neonates compared to those without BPD (13.1% vs. 21.2%, p = 0.01).

**Table 2:** Comparison of the studied neonates by their bronchopulmonary dysplasia (BPD) status. Characteristics data are presented by mean ± SD or by n (%). *Significant; **independent t-test was used; otherwise, chi-square test was used for statistical analysis.

Characteristics	Description	BPD** (n= 183)	No BPD (n= 1370)	St. test value	P
Sex	Male	106 (57.9)	713 (52.0)	2.24	0.14
	Female	77 (42.1)	657 (49.0)	2.24	0.14
Gestational age	≤ 28 weeks	129 (70.5)	495 (36.1)	79.2	
	> 28 weeks	54 (29.5)	875 (63.9)	79.2	
	< 750	59 (32.2)	178 (13.0)	128.5	
Birth weight (gm)	750-<1000	63 (34.4)	209 (15.3)	128.5	
	1000- <1250	37 (20.2)	251 (18.3)	128.5	
	1250-1500	14 (7.7)	276 (20.1)	128.5	
	>1500	10 (5.5)	456 (33.3)	128.5	
Apgar Score	Apgar 1 (mean ± SD) **	4.9 ± 2.0	5.7 ± 2.1	-4.60	
	Apgar 5 (mean ± SD) **	7.2 ± 1.5	7.7 ± 1.6	-3.05	0.002*
	Synchronized intermittent mandatory ventilation (SIMV)	131 (71.6)	582 (42.5)	85.4	
Mode of ventilation	Non-invasive ventilation (NIV)	22 (12.0)	659 (48.1)	85.4	
	High frequency oscillatory ventilation (HFOV)	30 (16.4)	129 (9.4)	85.4	
	Sepsis	84 (46.0)	229 (16.7)	85.4	
	Patent Ductus arteriosus (PDA)	62 (33.9)	168 (12.3)	59.8	
Medical conditions	Necrotizing enterocolitis	19 (10.4)	38 (2.8)	26.4	
	Periventricular leukomalacia (PVL)	8 (4.4)	33 (2.4)	2.42	0.12
	Intraventricular hemorrhage (IVH)	15 (8.2)	74 (5.4)	2.33	0.13
	Retinopathy of prematurity (ROP)	39 (21.3)	48 (3.5)	96.8	
	No	44 (24.0)	1019 (74.4)	190.2	
Blood transfusion	Complete blood	81 (44.3)	200 (14.6)	190.2	
	Platelets	34 (18.6)	95 (6.9)	190.2	
	Fresh frozen plasma (FFP)	24 (13.1)	56 (4.1)	190.2	
Hospitalization and patient outcomes	Length of hospital stay (days) mean ± SD	107 ± 48.0	33.2 ± 31.4	20.2	
	Death	24 (13.1)	291 (21.2)	6.59	0.01*

Table 3 presents the results of the univariate logistic regression analysis examining the association between blood transfusion and BPD. The analysis shows a significant association, with neonates receiving blood transfusions being 9.1 times more likely to have BPD (OR = 9.1, 95% confidence interval (CI): 6.3-13.1, p < 0.0001). When analyzing the type of blood transfusion product, it was found that neonates who received complete blood were 9.4 times more likely to have BPD (OR = 9.4, 95% CI: 6.3-13.4, p < 0.0001), those who received platelets had an 8.2-fold increased likelihood of having BPD (OR = 8.2, 95% CI: 5.1-13.5, p < 0.0001), and neonates who received fresh frozen plasma were 9.9 times more likely to have BPD (OR = 9.9, 95% CI: 5.6-17.4, p < 0.0001).

**Table 3:** Univariate logistic regression analysis for the association of bronchopulmonary dysplasia (BPD) and blood transfusion among the studied neonates. *Significant

Blood transfusion variables	Description	BPD (n= 183)	No BPD (n= 1372)	P	OR (95% CI)
Blood transfusion	No	33	1019	-	1.00 (Ref.)
	Yes	139	351		9.1 (6.3-13.1)
	No	44	1019	-	1.00 (Ref.)
Blood transfusion product	Complete blood	81	200		9.4 (6.3-13.4)
	Platelets	34	95		8.2 (5.1-13.5)
	Fresh Frozen Plasma (FFP)	24	56		9.9 (5.6-17.4)

Table 4 presents the results of the multivariate logistic regression analysis for the association between blood transfusion and BPD in neonates. After adjusting for potential confounders including gestational age, birth weight, Apgar scores, mode of ventilation, sepsis, PDA, NEC, and ROP, it was found that neonates who received blood transfusions were 3.8 times more likely to develop BPD compared to those who did not receive transfusions (OR = 3.8, 95% CI: 2.5-5.8, p < 0.0001). When examining the type of blood transfusion product, the results remained significant for all categories. Neonates who received complete blood had 4.1 times higher odds of developing BPD (OR = 4.1, 95% CI: 2.6-6.4, p < 0.0001), those receiving platelets had 3.2 times higher odds (OR = 3.2, 95% CI: 1.8-5.7, p < 0.0001), and neonates who received FFP had 3.9 times higher odds (OR = 3.9, 95% CI: 2.1-7.5, p < 0.0001).

**Table 4:** Multivariate logistic regression analysis for the association of BPD blood transfusion among the studied neonates. *Significant; **bronchopulmonary dysplasia OR is adjusted by gestational age categories, birth weight categories, Apgar1, Apgar5, mode of ventilation, sepsis, patent ductus arteriosus (PDA), necrotizing enterocolitis (NEC), and retinopathy of prematurity (ROP).

Blood transfusion variables	Description	BPD (n= 183)	No BPD (n= 1372)	P	OR^a^ (95% CI)
Blood transfusion	No	44	1019	-	(Ref.)
	Yes	139	351		3.8 (2.5-5.8)
	No	44	1019	-	1.00 (Ref.)
Blood transfusion product	Complete blood	81	200		4.1 (2.6-6.4)
	Platelets	34	95		3.2 (1.8-5.7)
	Fresh Frozen Plasma (FFP)	34	56		3.9 (2.1-7.5)

Table 5 presents the results of the stepwise regression analysis used to identify the predictors of BPD among the studied neonates. Blood transfusion emerged as the strongest predictor with an OR of 4.5, indicating that neonates who received a transfusion were significantly more likely to develop BPD. ROP and PDA were also identified as significant predictors, with ORs of 3.8 and 1.9, respectively. Sepsis was found to be another important predictor, with an OR of 1.7. In addition, NIV was associated with a significant reduction in the likelihood of developing BPD, with an OR of 0.43 (95% CI = 0.26-0.73). 

**Table 5:** Predictors of BPD among the studied neonates: the results of the stepwise regression analysis. *Significant

Predictive variables	P	OR	95% CI
Blood transfusion		4.5	2.91-6.70
Retinopathy of prematurity (ROP)	0.001*	3.8	2.31-6.43
Patent ductus arteriosus	0.001*	1.9	1.30-2.92
Sepsis	0.01*	1.7	1.22-2.53
Non-invasive ventilation (NIV)	0.03*	0.43	0.26-0.73

## Discussion

The current study reports an 11.8% incidence of BPD with the global incidence ranging from 10% to 89% [23]. Blood transfusions are a common and life-saving intervention for premature neonates [24], with previous studies suggesting that RBC transfusions may play a role in the development of BPD. In our study, 31.6% of the 1553 neonates received blood transfusions, with 76% of BPD cases receiving transfusions compared to 25.6% of non-BPD cases (p < 0.0001). A better understanding of the risk factors for BPD is a key step toward the prevention and adequate management of this disease [25].

Our findings indicate that blood transfusions significantly increased the risk of BPD, with transfusion recipients being 9.1 times more likely to develop the condition (OR = 9.1, 95% 95% CI: 6.3-13.1, p < 0.0001). This is consistent with other studies; for instance, Zhang et al. [26] in Guangdong found that neonates weighing less than 1500 g and receiving more than three RBC transfusions had a markedly higher risk of developing BPD (OR 10.2; 95% CI 2.1-47.6, n = 129). A recent meta-analysis of 21 studies including 6,567 healthy controls and 1,476 BPD patients found a significant association between RBC transfusion and BPD. The pooled unadjusted OR was 4.01 (95% CI: 2.31-6.97), while the adjusted OR was 5.11 (95% CI :3.11-8.4) [27]. 

In our study, the adjusted model showed a reduced risk compared to the unadjusted model, highlighting the impact of controlling for potential confounders. In the unadjusted model, neonates receiving transfusions were 9.1 times more likely to develop BPD (95% CI: 6.3-13.1). After adjusting for factors such as gestational age, birth weight, Apgar scores, ventilation method, sepsis, PDA, NEC, and ROP, the OR decreased to 3.8 (95% CI: 2.5-5.8). This reduction suggests that the unadjusted model overestimated the association, underscoring the importance of considering confounders to avoid misleading conclusions about causal relationships. 

Stepwise regression analysis identified several key predictors of BPD in neonates, with blood transfusion emerging as the strongest predictor (OR = 4.5), indicating that transfusion recipients were significantly more likely to develop BPD. This finding was supported by Bolat et al. [28] in Turkey, which examined 246 extremely preterm infants and found that both the volume and frequency of RBC transfusions were significant predictors of BPD incidence and severity. Similarly, a retrospective cohort study of 625 neonates (102 with BPD), reported RBC transfusion as a strong predictor of BPD in multivariable logistic regression analysis (OR = 7.5, 95% CI: 2.8-20.2) [6]. This association may reflect the role of transfusions in worsening lung injury, potentially through mechanisms such as inflammation or oxygen toxicity [15,27-29]. Additionally, the developmental differences between neonatal and adult blood components suggest that transfused adult platelets and RBCs could be more pro-inflammatory in neonates than in adults, potentially contributing to the inflammatory processes associated with the development of BPD [12-14]. These findings further emphasize the importance of RBC transfusions as a critical factor in the pathogenesis of BPD and highlight the need for careful monitoring of transfusion practices in preterm neonates [15,27]. 

ROP and PDA were significant predictors of BPD, with OR of 3.8 and 1.9, respectively, indicating that vascular complications in preterm infants can contribute to BPD. Sepsis, with an OR of 1.7, was also identified as an important predictor, likely due to its inflammatory effects, which can exacerbate lung damage and increase the risk of BPD. These findings align with previous studies [27,28,29]. By contrast, NIV was found to be a protective factor, with an OR of 0.43 (95% CI = 0.26-0.73), indicating that its use significantly reduces the likelihood of developing BPD. Similarly, Keszler and Sant'Anna reported that effective use of non-invasive respiratory support lowers the risk of lung injury. This suggests that NIV may help minimize the lung trauma associated with invasive mechanical ventilation, thus reducing the risk of BPD [30].

Moreover, a recent study's sensitivity analyses indicated that noncompliance with the new restrictive guidelines could contribute to the development of BPD with 57% of RBC transfusions and 68% of platelet transfusions not adhering to the guidelines. Modeling predictions suggested that adhering to these guidelines could reduce the transfusion rate by 20%-30% and decrease the incidence of moderate to severe BPD by approximately 4-6% [15]. 

This study has several strengths that enhance its validity and relevance. The large sample size of 1553 neonates strengthens the statistical power of the findings and makes the results more generalizable. The study also benefits from comprehensive data collection, including a wide range of clinical variables such as gestational age, birth weight, and neonatal conditions, which allowed for a thorough examination of the association between blood transfusion and BPD. In addition, the use of both univariate and multivariate logistic regression analyses provides a detailed understanding of the relationship, with the multivariate analysis controlling for potential confounders. The study's long duration, spanning from 2011 to 2020, helps capture trends and variations in clinical practices over time, further increasing the robustness of the findings. Furthermore, the clinical relevance of the study is significant, as BPD is a common and serious condition in preterm infants, and understanding factors associated with its development, such as blood transfusion, is crucial for neonatal care.

However, there are several limitations to consider. The retrospective design of the study relies on existing medical records limiting the ability to establish causal relationships. Moreover, there may be unmeasured factors that influence both blood transfusion and the development of BPD, which could impact the results. The study was conducted at a single center, which may limit its generalizability to other institutions or populations with different practices. In addition, potential variations in transfusion protocols over the 10-year study period might influence the observed relationship between transfusion and BPD as well as limit the ability to establish causality and generalize the findings. Furthermore, the timing of transfusions (e.g., early vs. late), which could potentially impact the risk of BPD, was not captured in the study.

## Conclusions

This study highlights a significant association between blood transfusion and an increased risk of developing BPD in neonates, with transfusion recipients being more likely to develop the condition. However, the multivariate results emphasize the complex interaction of factors such as transfusions, cardiovascular and retinal complications, infections, and respiratory support strategies that collectively influence the risk of BPD. Addressing these factors through early detection and targeted interventions could help reduce BPD incidence and improve outcomes for preterm neonates. Future prospective studies involving multiple centers with standardized transfusion protocols are needed, which will also help inform clinical practices in neonatal care.
